# Hemorrhagic adenovirus cystitis in a newborn

**DOI:** 10.1515/crpm-2022-0018

**Published:** 2022-12-30

**Authors:** Susana M. D. Alexandre, Carolina O. C. C. Matos, Fabiana C. F. Fortunato, Ana R. M. C. Sandes

**Affiliations:** Serviço de Pediatria – Centro Hospitalar do Oeste, Caldas da Rainha, Portugal; Unidade de Nefrologia e Transplantação Renal Pediátrica, Departamento de Pediatria, Hospital de Santa Maria – Centro Hospitalar Universitário Lisboa Norte, Lisbon, Portugal

**Keywords:** adenovirus, cystitis, hematuria, nephrology, newborn

## Abstract

**Objectives:**

Gross hematuria is rare in the neonate and requires prompt etiology evaluation and intervention. This article aims to draw attention to adenovirus as a cause of hematuria in newborns.

**Case presentation:**

We present the case of a newborn admitted to the neonatal unit after birth with respiratory distress. Empiric treatment with ampicillin and gentamicin was initiated. He presented a favorable clinical and laboratory course with decreasing inflammatory parameters. On day 7 gross hematuria was detected and the urinalysis revealed red blood cells, trace of proteins and leukocytes. Cefotaxime was added after urine and blood cultures. Doppler ultrasound showed bladder sediment with no signs of renal venous thrombosis and the cultures were negative. There was a progressive improvement of gross hematuria with resolution on day 16. Urine adenovirus PCR was positive and the diagnosis of adenovirus hemorrhagic cystitis was made.

**Conclusions:**

Adenovirus should be considered as a potential etiology if clinical symptoms and urinalysis are suggestive of infection, but the urine culture is negative and ensuring that all other possible causes of hematuria are ruled of. As far as the authors know, this is the first case report of a newborn with adenovirus hemorrhagic cystitis.

## Introduction

Neonatal hematuria, while uncommon, can be a symptom of a serious disease. Gross hematuria should be evaluated promptly as several causes are associated with renal injury, including thrombotic events, infections, acute tubular or cortical necrosis, and glomerulonephritis [1]. Also some uropathies can be the cause of gross hematuria. There is no recent data approaching the incidence of neonatal hematuria. A retrospective study of neonates with gross hematuria over a 17-year period between 1950 and 1967 described an incidence of 0.21 per 1,000 admissions in newborns. Renal vein thrombosis, polycystic kidney and obstructive uropathies comprised about two thirds of hematuria causes [2].

Hematuria is an uncommon presentation of a neonatal urinary tract infection (UTI), however, the diagnosis should be considered to prevent associated morbidity [1]. Neonates with urosepsis with organisms other than *Escherichia coli* are at increased risk of having structural abnormalities, including vesicoureteral reflux. Hematuria due to adenovirus and human polyomavirus virus cystitis has been reported in immunocompetent children, but there are no published reports in the neonatal period [3]. As far as the authors know, this is the first case report of a newborn with adenovirus hemorrhagic cystitis.

## Case presentation

We report a case of a male newborn, second child of a healthy mother. The pregnancy was uneventful and he was born at 39 weeks’ gestation by a vacuum extraction after a 19 h spontaneous membrane rupture. During labor the mother presented with fever (38.1 °C), leukocytosis with neutrophilia and elevation of C-Reactive protein. Antibiotics were started. Apgar scores were 8, 8 and 9 at 1, 5 and 10 min, respectively. After birth, the 4.1 kg newborn was transferred to the neonatal special care unit with respiratory distress. He needed supplemental oxygen for the first 8 h, after which he showed significant clinical improvement. Despite clinical improvement the blood panel at 24 h of life showed elevated inflammatory markers (C-Reactive Protein 4.6 mg/dL and procalcitonin 10.79 ng/mL) and treatment with ampicillin and gentamicin was initiated. Blood cultures (day 1 and 7) were sterile.

At day 7 he presented with gross hematuria (bright red urine) with a normal clinical exam. He had no hypertension, oliguria or edema. Urine microscopy showed a high number of red and white blood cells and trace proteins (25 mg/dL). Serum creatinine and coagulation were normal. Although there was no leukocytosis or elevation of C-Reactive Protein, hematogenous bacterial pyelonephritis was suspected and cefotaxime was added. Urine and blood cultures were sterile. Renal ultrasound (day 10) showed an enlarged left kidney, with a slight hyperechogenicity of renal sinus fat, and an echogenic content inside the bladder, suggesting an infectious process (cystitis and possibly pyelonephritis). The Doppler ultrasound (only possible at day 15) did not have indirect signs of renal venous or arterial thrombosis. He completed 8 days of gentamicin, 14 days of ampicillin and 9 days of cefotaxime.

Gross hematuria persisted until day 16 and urine microscopy on day 15 still showed an elevated number of red (250/µL) and white blood cells (positive leukocyte esterase test +++), as well as a low level of proteinuria (25 mg/dL) and occasional granular casts. Nitrite test was negative. A new urine culture was also sterile. Urine DNA by polymerase chain reaction (PCR) for adenovirus was positive, cytomegalovirus and human polyomavirus were negative. The newborn remained clinically stable and was discharged on day 18 with a normal urinalysis.

On 1 month follow-up visit he remained asymptomatic, with normal urine microscopy. He had positive anti-adenovirus antibodies (IgG) and normal renal ultrasound. After one month he remained asymptomatic, with normal urine microscopy and normal renal ultrasound.

## Discussion

Neonatal gross hematuria is rare and requires careful evaluation. We describe the case of a one week old newborn presenting with gross hematuria during a course of antibiotics for early-onset neonatal sepsis.

Gross hematuria initial assessment implies confirmation of true hematuria with an urinalysis. Urate crystals, diaper rash or rectal bleeding can be misdiagnosed as gross hematuria. In myoglobinuria and hemoglobinuria, urinalysis will be positive for blood but urine microscopy will be negative for RBCs [3]. In our case urine microscopy confirmed hematuria. Unfortunately it was not possible for our laboratory to characterize de morphology of red blood cells, namely searching for dysmorphic erythrocytes. Nevertheless, the red bright urine color and the absence of significative proteinuria were against a glomerular origin for the hematuria.

He had no apparent risk factors, such as procedures (umbilical lines or bladder catheterization), trauma or medication associated with hematuria [4]. Blood tests didn’t show anomalies in coagulation or increased inflammatory parameters. The kidney and bladder ultrasound showed signs suggestive of an infectious process (cystitis and possibly pyelonephritis), so, despite innocent blood screening, we considered an UTI that was not responding to ampicillin and gentamicin, and cefotaxime was added. Vascular thrombosis was also an initially considered hypothesis. Both renal vein thrombosis and artery thrombosis can present with gross hematuria. Renal vein thrombosis is more common and occurs especially in males, it is usually on the left side and early-onset sepsis represents an additional risk in this case [3]. However, the normal serum creatinine value and the Doppler ultrasound result ruled out this diagnosis.

Our patient had 8 days of macroscopic hematuria and image evidence of UTI, in spite of antibiotic therapy. However, the benign clinical and laboratory course made multidrug-resistant bacteria a less likely hypothesis. Therefore antibiotics were discontinued and viral agents were considered, like adenovirus, cytomegalovirus and human polyomavirus. Adenovirus DNA in the urine was positive.

Adenovirus are known agents of upper respiratory, gastrointestinal, and conjunctival infections in healthy children. However, their pathogenicity is altered by the host immune status [5]. There are a limited number of adenovirus infection reports in young infants, but published case series indicate that adenovirus can cause serious, life-threatening disease in the neonate [6, 7].

Adenovirus is also commonly associated with acute hemorrhagic cystitis, mainly serotypes 11, 21, 34 and 35 [8, 9]. Hemorrhagic cystitis is usually self-limiting (with hematuria lasting approximately 4 days), more common in young males where typically no treatment is necessary, except in severely immunocompromised patients. Urine detection of adenovirus on patients with hemorrhagic cystitis is pathognomonic of adenoviral cystitis [5, 8].

As far as we know, there are no published reports of adenovirus cystitis in a newborn [3]. This diagnosis was considered since every other etiologies were excluded. As in the case of older children, the case presented here had also a benign evolution.

Transmission of adenovirus to the neonate can occur either vertically from an infected mother or horizontally after birth [7]. Although most neonatal adenovirus infections are believed to be acquired in the birth canal, congenital infections have been described, presumably through transplacental transmission [6]. Our patient presented hematuria at an age of 7 days, so there is doubt about the transmission mechanism (we could not find any published data about UTI incubation period, but usually it is 2–14 days for adenovirus respiratory tract infection and 3–10 days for gastrointestinal infection) [10]. The lack of adenovirus testing in mother precluded precise knowledge of transmission mechanism.

This article aims to draw attention to adenovirus as a cause of hematuria in newborns. The importance lies in differentiating acute hemorrhagic cystitis from other more serious diseases presented with hematuria [11], namely vascular changes, such as renal vein/arterial thrombosis, uropathies, glomerulopathies and coagulation disorders. This is an exclusion diagnosis. Thus, adenovirus should be considered as a potential etiology if clinical symptoms and urinalysis are suggestive of infection, but the urine culture is negative1 [12] and ensuring that all other possible causes of hematuria are ruled of.

## References

[1] Jernigan SM (2014). Hematuria in the newborn. Clin Perinatol.

[2] Emanuel B, Aronson N (1974). Neonatal hematuria. Am J Dis Child.

[3] Joseph C, Gattineni J (2016). Proteinuria and hematuria in the neonate. Curr Opin Pediatr.

[4] Young TE, Mangum B (2020). Neofax.

[5] Paduch DA (2007). Viral lower urinary tract infections. Curr Urol Rep.

[6] Gleason CA, Juul SE (2018). Avery’s diseases of the newborn.

[7] Ronchi A, Doern C, Brock E, Pugni L, Sánchez PJ (2014). Neonatal adenoviral infection: a seventeen year experience and review of the literature. J Pediatr.

[8] Bernstein D (2016). Nelson textbook of pediatrics.

[9] Flomenberg P, Kojaoghlanian T, Post TW Pathogenesis, epidemiology, and clinical manifestations of adenovirus infection. ..

[10] Allen UD, Demmler-Harrison GJ, Long SS, Prober CG, Fischer M (2017). Adenoviruses. Principles and practice of pediatric infectious diseases.

[11] Mufson MA, Zollar LM, Mankad VN, Manalo D (1971). Adenovirus infection in acute hemorrhagic cystitis. Am J Dis Child.

[12] Boyer OG., Post TW Evaluation of gross hematuria in children. ..

